# Antibiofilm Activity and Synergistic Effects of Thymol-Loaded Poly (Lactic-Co-Glycolic Acid) Nanoparticles with Amikacin against Four *Salmonella enterica* Serovars

**DOI:** 10.1155/2023/7274309

**Published:** 2023-01-16

**Authors:** Christian Ramsès Kuaté Tokam, Borel Bisso Ndezo, Nathalie Boulens, Eric Allémann, Florence Delie, Jean Paul Dzoyem

**Affiliations:** ^1^Laboratory of Microbiology and Antimicrobial Substances, Department of Biochemistry, Faculty of Science, University of Dschang, Cameroon P.O. Box 67, Dschang, Cameroon; ^2^School of Pharmaceutical Sciences, University of Geneva, Geneva, Switzerland; ^3^Institute of Pharmaceutical Sciences of Western Switzerland, University of Geneva, Geneva, Switzerland

## Abstract

**Background:**

Salmonella species are frequently linked to biofilm-associated infections. Biofilm formation intensively reduces the efficacy of antibiotics and the host immune system. Therefore, new therapeutic strategies are needed. Thymol, the main monoterpene phenol found in *Thymus vulgaris*, has been shown to possess potent antibiofilm activity. Our previous findings showed that thymol enhanced the antibiofilm activity of aminoglycosides against *Salmonella enterica* serovars. However, the clinical potential of thymol has not yet been realized due to its low aqueous solubility and high volatility. Nano-based drug delivery systems have emerged as a novel strategy to resolve these problems. This study aimed to investigate the antibiofilm activity of thymol-loaded poly (lactic-co-glycolic acid) nanoparticles (TH-NPs) and their synergism when used in combination with amikacin antibiotics.

**Methods:**

The antibacterial activity of TH-NPs was evaluated using the broth microdilution method. Biofilm formation and antibiofilm assays were performed by the miniaturized microtiter plate method. Interaction studies between TH-NPs and amikacin against biofilm were determined using the checkerboard method.

**Results:**

TH-NPs exhibited antibacterial activity against planktonic cells of *S. enterica* serovars that were more efficient (8 to 32 times) than free thymol alone. *S.* Typhimurium and *S.* Choleraesuis isolates were considered strong biofilm producers. The combination of TH-NPs with amikacin showed synergistic activity in the inhibition and eradication of *S. enterica* serovar biofilm. The minimum biofilm inhibitory concentration (MBIC) and minimum biofilm eradication concentration (MBEC) of amikacin were reduced by 32 to 128-fold when used in combination with TH-NPs. Time-kill kinetic studies showed that the combination of TH-NPs with amikacin possesses bactericidal action.

**Conclusion:**

This study suggests that the combination of TH-NPs with amikacin can be an alternative to overcome biofilm-associated*Salmonella* diseases and therefore should be further explored as a model to search for new antibiofilm drugs.

## 1. Introduction

*Salmonella* is a genus of the family Enterobacteriaceae. Taxonomically, this genus is separated into two species: *Salmonella bongori* and *Salmonella enterica*, which contain six subspecies with serovars like *Salmonella* Typhi, *Salmonella* Enteritidis, *Salmonella* Paratyphi, *Salmonella* Typhimurium, and *Salmonella* Choleraesuis [1].*Salmonella enterica* causes typhoidal and nontyphoidal salmonellosis (NTS) [2]. The WHO estimates the global typhoid fever disease burden at 11–20 million cases annually, resulting in about 128 000–161 000 deaths per year [3]. On the other hand, the global human health impact of nontyphoidal *Salmonella* is high, with an estimated 93.8 million cases, of which an estimated 80.3 million are food-borne, and 155.000 deaths each year [4]. Salmonellosis is an important source of morbidity [5]. Their clinical picture varies from a common gastroenteric to enteric fevers, which are life-threatening, requiring prompt and correct antibiotherapy [6]. However, resistance to antibiotics is increasing. One of the mechanisms of resistance used by *Salmonella* is biofilm formation, which also appears as a potent virulence factor [7]. A biofilm is defined as a sessile microbial community in which cells are attached to a surface or other cells and embedded in a protective extracellular polymeric matrix. This mode of growth exhibits altered characteristics, especially regarding gene expression and protein production [8]. The infections associated with biofilms are difficult to treat because of the slow penetration and/or sequestration of antimicrobial agents into the biofilm through the extracellular matrix, the presence of multidrug-resistant persister cells, and the low metabolic state at the base of the biofilm [9]. Thereby, biofilms formed by bacteria are more resistant to antimicrobials than nonadherent cells with minimum eradication concentrations 10 to 1000 times higher than planktonic cells [10]. Therefore, there is an urgent need for the identification of new approaches and therapeutic formulations that target the biofilm mode of growth to prevent or treat biofilm-related infections. Drug combinations have been used as an alternative to effectively combat biofilm. Antibiotic-antibiotic combination and association of an antibiotic with bioactive natural products are interesting approaches to fight biofilms.

Natural products, such as thymol and their structural analogs, have historically made a major contribution to pharmacotherapy [11]. Thymol has been applied as a spice and antibacterial product. In our recent study, thymol has shown antibiofilm activity and synergistic effects with aminoglycoside antibiotics against *Salmonella spp* and *Klebsiella spp* [12, 13]. However, the use of thymol in the pharmaceutical field has recently been limited by its poor water solubility, stability, and high volatility [14]. To overcome this limitation, thymol can be smartly encapsulated into a nanocarrier [14, 15]. Among the different polymers used for nanoparticle preparation, copolymers of poly (lactic-*co*-glycolic acid) (PLGA) are frequently used for their good properties as used in this study. PLGA is a US Food and Drug Administration (FDA)-approved copolymer for human use. It is widely exploited in the design of nanoparticles because of its biodegradability and biocompatibility.

Furthermore, as nanoparticles, it protects drug molecules from degradation and aids in producing sustained and targeted delivery [16]. A previous study reported a strong antibacterial activity of TH-NPs against planktonic cells of *Escherichia coli* and *Staphylococcus aureus* [17]. We have recently demonstrated a remarkable synergistic antibiofilm effect of thymol in combination with aminoglycoside antibiotics against *S. enterica* serovars. Thymol was shown to potentiate the antibiofilm activity of amikacin, kanamycin, and streptomycin, and we recorded the amikacin-thymol combination as the most effective one [12, 13]. Therefore, we hypothesized that the previously observed antibiofilm and synergistic effects of thymol might be improved by the encapsulation of thymol into nanoparticles. The present study aimed to investigate the combination of TH-NPs with amikacin against the biofilm of *S. enterica* serovars.

## 2. Materials and Methods

### 2.1. Chemicals and Naturals Products

Amikacin disulfate salt (Acros Organics) as well as, thymol (purity ≥98.5%), Safranin, *p*-iodonitrotetrazolium (INT) chloride, and safranin were purchased from Sigma-Aldrich. PLGA (RESOMER RG 503 H, 50 : 50, Mw 24000–38000) was obtained from Boehringer Ingelheim Pharma GmbH&Co. Polyvinyl alcohol (PVAL) was obtained from Roche Diagnostics.

### 2.2. Nanoparticle Preparation

TH-NPs were prepared using the single emulsion-solvent evaporation method previously described with slight modifications [15]. Briefly, 75 mg of PLGA and 7.5 mg of thymol were dissolved in 1.5 mL of dichloromethane. Then, 3 mL of polyvinyl alcohol solution prepared in distilled water at 2% (W/V) was added dropwise into the organic phase and magnetically stirred for 3 h to evaporate dichloromethane. The solution was centrifuged at 65,000 ×g for 20 min, and the obtained suspension was washed thrice with distilled water. The TH-NP sample suspension obtained was freeze-dried using a Christ Alpha 2–4 LDplus freeze dryer (Christ, Osterode am Harz, Germany) and stored at −20°C for further analysis.

Dynamic light scattering (DLS) was used to measure the particle size, polydispersity index (PDI), and *zeta* potential of the obtained nanoparticles.

### 2.3. Microorganisms and Growth Conditions

The *S*. Typhi (ATCC 6539) reference strain used in this study was purchased from the American Type Culture Collection (ATCC). Four clinical isolates of *Salmonella enterica* serovars, *S*. Enteritidis, *S*. Typhi, *S*. Typhimurium, and *S*. Choleraesuis, were all provided by “Centre Pasteur du Cameroun.” All of them were plated from cultures, which were stored at –80°C onto Salmonella-Shigella agar (SSA) (Condalab) for 18–24h at 37°C. Cultures were subsequently subcultured and maintained onto Muller–Hinton agar (MHA, Sigma-Aldrich) plates at 4°C until needed for further bioassay.

### 2.4. Determination of the Minimum Inhibitory Concentration (MIC) and Minimum Bactericidal Concentration (MBC) of TH-NPs

The MIC test was performed in a sterile 96-wellround-bottom microtiter plate using standard broth microdilution methods, while the MBC test was performed on MHA plates. For the MIC test, first, the 96-well microtiter plates were only filled with 100 *μ*L of MHB. After that, 100 *μ*L of TH-NP stock solution (4096 *μ*g/mL) was added and diluted twofold. Concentrations of TH-NPs ranged from 0.5 to 1024 *μ*g/mL. 100 *μ*L of bacteria at 1.5 × 10^6^ CFU/mL (with 7.5 × 10^5^ as the final concentration) were added into the wells. The selected wells filled with MHB only and MHB and organism, to serve as negative and positive controls, respectively. The microtiter plates were then incubated at 37°C for 24 h, after which 40 *μ*L of *p*-iodonitrotetrazolium (INT) chloride at 0.2 mg/mL was introduced into each well, and observations were made for the yellow dye to pink coloration after 30 min of incubation at 37°C, which signifies growth. The MIC was recorded as the lowest concentration that prevented the color change in the medium. The experiment was carried out in triplicate [18].

The minimum bactericidal concentration (MBC) was defined as the lowest concentration of antibacterial agents that completely kill organisms. This test was performed by plating the suspension from each well of the microtiter plates into an MHA plate. After that, the plates were incubated at 37°C for 24 h. The lowest concentration with no visible growths on the MHA plate was taken as the MBC value [19].

### 2.5. Ability of Biofilm Formation

A microtiter plate (MtP) assay was used to determine and quantify biofilm production by using a microplate reader. Based on the kinetic metabolic activity in our previous work, the biofilm biomass of *S. enterica* serovars was determined with a colorimetric method based on safranin dye. 48 hours has been chosen as the training time [12]. Briefly, 100 *μ*L of a bacterial suspension at 1.5 × 10^6^ CFU/mL was added to 100 *μ*L of MHB supplemented with 2% glucose into the 96-wellflat-bottomed sterile polystyrene microplates and incubated at 37°C for 48 h [20]. 2% glucose was added to MHB to increase biofilm formation [21]. After incubation, planktonic cells in the well of the microplate were discharged by washing twice with 300 *μ*L of phosphate-buffered saline (PBS) at 7.2 pH. After that, step biofilms were fixed with 150 *μ*L of methanol for 20 min at room temperature. To perform biofilm formation, 150 *μ*L of 1% (w/v) safranin 1% was used to stain biofilms. After 15 min of incubation at room temperature, excess safranin was removed, and the dye bound to the adherent cells was solubilized with 150 *μ*L of 95% ethanol. Optical density was measured spectrophotometrically at 570 nm by using a microplate (SpectraMax® ix 3, Molecular Devices). The studies were repeated in triplicate. Uninoculated wells containing sterile MHB supplemented with 2% glucose were considered negative controls and were used as blanks. The blank absorbance values were used to identify whether biofilm formation of *S. enterica* serovars exists or not. The wells of isolates whose ODi (OD isolate) values were higher than those of blank wells were all considered to be biofilm producers. The cutoff value (ODc) can provide categorization of isolates as biofilm producers or not. ODc = average OD of the negative control + (3 × standard deviation (SD) of the negative control); ODi = average OD of the isolate − ODc. A negative value obtained from this formula and represented as zero indicates a lack of biofilm production, whereas a positive value indicates biofilm production. Based on OD of the isolate (ODi), interpretations can be performed as follows: ODi ≤ ODc no biofilm production (NBP), ODc ˂ ODi ≤2 × ODc weak biofilm production (WBP), 2 × ODc ˂ OD ≤ 4 × ODc moderate biofilm production (MBP), and 4 × ODc ˂ ODi strong biofilm production (SBP) [22].

### 2.6. Antibiofilm Assay

#### 2.6.1. Inhibition of Bacterial Attachment

The effect of TH-NPs on the biofilm formation of *S. enterica* serovars was evaluated as described by Ahmed et al., with minor modifications [23]. The assay was performed in a sterile 96-well polystyrene plate. Briefly, 100 *μ*L of bacterial inoculum (1.5 × 10^6^ CFU/mL) was inoculated and cultured with or without 100 *μ*L of TH-NPs (at concentrations ranging from 0.5 to 1024 *μ*g/mL), without shaking at 37°C. After 24 h incubation, planktonic cells were removed by washing each sample three times in sterile phosphate buffer solution (PBS) at pH 7.2, and adherent cells were fixed with methanol for 20 min at room temperature. Then, the plates were treated as described above in the biofilm formation assay [12]. Wells containing MHB supplemented with glucose 2% without bacteria were used as the negative control and taken as the blank, whereas wells containing MHB supplemented with glucose 2% with bacteria were used as the positive control. The experiment was carried out in triplicate and repeated three times. The percentage inhibition of biofilm biomass is calculated as follows:

(1) $$\begin{array}{c} \%nhibition\;biofilm\;biomass=\frac{\left({OD}_{ontrol}-{OD}_{blank}\right)-\left({OD}_{test}-{OD}_{lank}\right)}{\left({OD}_{control}-{OD}_{blank}\right)}\;x\;100 \end{array}$$

The minimum biofilm inhibitory concentration (MBIC) was defined as the lowest concentration of TH-NPs that inhibit biofilm biomass by 100%.

#### 2.6.2. Biofilm Eradication Assay

Eradication of biofilm by TH-NPs was carried out according to the protocol described by Kirmusaoaylu and Kalşkçl [20]. After biofilm formation for 48 h, the medium was gently discarded. To remove nonadherent bacterial cells, the wells were carefully washed with PBS. Then, the plates were filled with 100 *μ*L of MHB supplemented with 2% glucose and 100 *μ*L of TH-NPs at concentrations ranging from 1 to 2048 *μ*g/mL. The plates were incubated at 37°C for 24 h. After that, the plates were treated as previously described for the biofilm inhibition assay. The minimum biofilm eradication concentration (MBEC) was defined as the lowest concentration of TH-NPs reducing biofilm biomass in the preformed biofilm by 100% and calculated as previously described. The experiment was performed in triplicate and repeated three times.

### 2.7. Combination Studies

#### 2.7.1. TH-NPs in Combination with Amikacin against Biofilm Formation

The checkerboard method was performed to determine the effect of combining TH-NPs and amikacin on the biofilm formation of *S. enterica* serovars. By using MHB supplemented with 2% glucose, TH-NPs were serially diluted across each row of the 96-well microtiter tray, and serial dilutions of antibiotics were then added to each column. Therefore, each row and column contained a fixed concentration of one agent and decreasing concentration of the other agent. Each tray contained one row and one column, with serial dilution of each agent alone. The remainder of the assay was performed as described for the broth microdilution assay. Then, 100 *μ*L of the bacterial inoculum (1.5 × 10^6^ CFU/mL) was added to each well, and the plates were incubated at 37°C for 24 h. The final concentrations ranged from 1 to 64 *μ*g/mL for TH-NPs and from 15625 × 10^−6^ to 16 *μ*g/mL for amikacin. After incubation, the broth in the wells was gently removed, and the plates were washed three times with PBS. Untreated wells were used as the positive control, and wells containing MHB without bacteria were used as the blank. The minimum biofilm inhibitory concentration (MBIC) of each compound was determined. The effect of combinations was determined by calculating the fractional inhibitory concentration index (FICI) by using the following equation: FICI = (MBIC of TH-NPs in the combination/MBIC of TH-NPs alone) + (MBIC of amikacin in combination/MBIC of amikacin alone). The type of interaction was defined based on the FICI value as follows: synergy when FICI ≤ 0.5, additivity when 0.5 ˂ FICI≤1, indifference when 1 ˂ FICI ≤ 4, and antagonism when FICI ˃ 4 [24].

#### 2.7.2. TH-NPs in Combination with Amikacin against Preformed Biofilm

To evaluate the effect of the combination of TH-NPs with amikacin to eradicate the biofilm of the same strains previously used, the checkerboard method was used as described by Hu et al., with slight modifications [25]. After biofilm formation for 48 h, planktonic cells were gently removed, and the plate was washed three times with PBS at pH 7.2. Then, 100 *μ*L of MHB supplemented with 2% glucose and 50 *μ*L of each substance as described above were added to adherent cells into the wells at a final concentration of 1 to 64 *μ*g/mL for TH-NPs and 15625 × 10^−6^ to 16 *μ*g/mL for amikacin. The wells containing medium and bacteria were used as negative and positive controls, respectively.

After incubation at 37°C for 24 h, the minimum biofilm eradication concentration (MBEC) was then determined. The FICI was calculated and interpreted as described above.

### 2.8. Kinetic Studies of Biofilm Biomass

The combination of TH-NPs with amikacin was used to assess the prevention of biofilm biomass and disruption of mature biofilm biomass. This assay was examined by using a 96-well plate (Flat bottom, Sterile; Corning, USA) as previously described with slight modifications [26]. Concerning kinetics of the inhibition of biofilm formation, first, biofilm was incubated and treated at 37°C at six-time points 4, 8, 12, 16, 20, and 24 h under static conditions. After that, planktonic cells were removed, and the wells were gently washed thrice with PBS at 7.2 pH. Then, the plates were treated as previously described. Note that untreated wells were used as the positive control, and the wells containing MHB broth without bacteria were used as the blank. The microplate was measured spectrophotometrically at 570 nm by using a microplate reader.

Kinetic biofilm biomass disruption of the preformed biofilm was carried out as described by Kamble and Pardesi with some modifications [27]. 100 *μ*L of a bacterial suspension at 1.5 × 10^6^ CFU/mL of the 18 h old culture was added to wells of a flat-bottom96-well microplate containing 100 *μ*L of MHB supplemented with 2% glucose. The plates were incubated further at 37°C for 48 h to allow for biofilm formation. Then, media containing planktonic cells were removed from the wells, and the biofilm was washed thrice with PBS. Untreated wells containing bacteria and wells with MHB only were used as positive and negative controls, respectively. After treatment, TH-NPs and amikacin were added alone or in combination and incubated at six-time points 1.5, 3.0, 4.5, 6.0, 7.5, and 24 h at 37°C. After the plates were treated as mentioned above, absorbance was measured at 570 nm. In both cases, the experiments were conducted three times in triplicate.

### 2.9. Time-Kill Kinetic Assay

The time-kill kinetic assay was used to study the activity of combination of TH-NPs with amikacin against *Salmonella enterica* serovars and determine the bactericidal, bacteriostatic, or synergistic activity of this combination over time [28]. This assay was carried out by estimating surviving biofilm cells using the viable cell plate-counting method [29]. The kinetic against inhibition of biofilm biomass was conducted over a range of seven different times 0, 4, 8, 12, 16, 20, and 24 h, whereas the time-kill kinetic assay against disruption of preformed biofilm was performed at 0, 1.5, 3.0, 4.5, 6, 7.5, and 24 h. After each incubation period, media were gently discarded, and the plates were washed three times with PBS. Then, individual components of each combination, either TH-NPs and amikacin alone or in combination at a synergistic concentration effect, were introduced, and the plates were incubated at different time points mentioned above at 37°C. After that, adherent cells were suspended with PBS via scraping followed by serial dilution in sterile normal saline. Then, 100 *μ*L of each dilution sample was plated on MHA.

After incubation of the MHA plate at 37°C for 24 h, colonies were counted, and results were expressed as log_10_ CFU/mL. Bactericidal activity was defined as greater than or equal to 3 log_10_ − fold decrease in colony-forming units (surviving bacteria) compared with the positive control, whereas synergy was defined as ≥2 log_10_ decrease in colony-forming units at 24 h by combination of TH-NPs with amikacin in comparison with TH-NPs or amikacin alone [28].

### 2.10. Statistical Analysis

GraphPad Prism software version 8.0.1 was used to analyse the data obtained. The results were presented as means ± standard deviation (SD) of three independent experiments. Statistical significance between the treated and control groups was analysed through the one-way analysis of variance (ANOVA) at *p* value ˂0.05.

## 3. Results

### 3.1. Nanoparticle Preparation

We obtained nanoparticles of size around 192 nm with a polydispersity index of 0.09 and a *zeta* potential of −5.39 mV [15].

### 3.2. Antibacterial Activity of TH-NPs against Planktonic Cells of *S. enterica*

The results of the antibacterial activity of TH-NPs against planktonic cells of *S. enterica* serovars are presented in Table 1. TH-NPs were more active (4 to 16 times) against *S. enterica*, showing MIC and MBC values ranging from 4 to 8 *μ*g/mL and 4 to 16 *μ*g/mL, respectively, compared to free thymol where MIC values ranged from 64 to 128 *μ*g/mL and 128 to 256 *μ*g/mL for MBC values. It should be noted that MIC and MBC of thymol are data taken from the previous study and were used in this study only for comparison purposes [12].

### 3.3. Biofilm Formation

**Figure 1:**
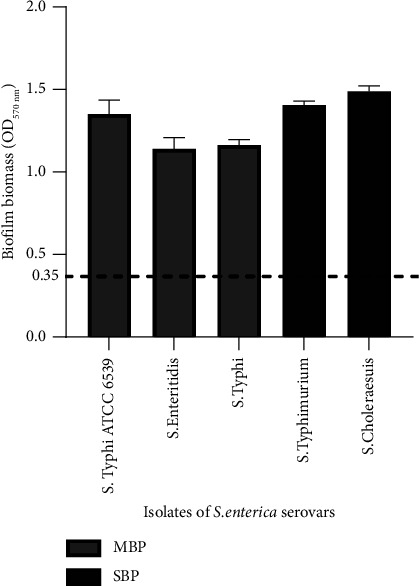
Quantification at 570 nm of biofilm biomass of *Salmonella enterica* serovars by staining with safranin 1%. MBP: moderate biofilm producer; SBP: strong biofilm producer. The dashed line represents the value of the optical density of control (OD_C_) at 570 nm.

Based on ODc = 0.35, our data revealed that all S. *enterica* isolates were biofilm producers*. S.* Typhimurium (1.40 ± 0.094) and *S.* Choleraesuis (1.49 ± 0.103) isolates were considered strong biofilm producers, while *S.* Typhi ATCC 6539 (1.35 ± 0.084), *S.* Enteritidis (1.14 ± 0.097), and *S.* Typhi (1.16 ± 0.034) isolates were classified as moderate biofilm producers (Figure 1).

### 3.4. Antibiofilm Effects of TH-NPs and Amikacin

#### 3.4.1. Antibiofilm Activity of TH-NPs Alone and in Combination with Amikacin against Biofilm Formation

The results of antibiofilm activity of TH-NPs and amikacin alone, and in combination against biofilm formation, are shown in Table 2. It was observed that MBIC values of TH-NPs in combination ranged from 4 to 8 *μ*g/mL. TH-NPs combined with amikacin exhibited synergistic (FICI = 0.13–0.28) antibiofilm activity against all isolates of *S. enterica* tested and reduced from 4 to 128 times the MBIC of amikacin.

#### 3.4.2. Antibiofilm Activity of TH-NPs Alone and in Combination with Amikacin against Preformed Biofilm

The result of antibiofilm activity of TH-NPs alone and in combination with amikacin against preformed biofilm is shown in Table 3. It was observed that the MBEC values of TH-NPs ranged from 64 to 128 *μ*g/mL.

TH-NPs showed synergistic action (FICI = 0.06–0.27) with amikacin against all mature biofilm of *S. enterica* serovars. MBEC of amikacin and TH-NPs was reduced 4 to 32 times and 32 to 128 times, respectively. Note that the results of amikacin alone recorded in this table come from our previous study [12].

### 3.5. Kinetic Study of Biofilm Biomass

**Figure 2:**
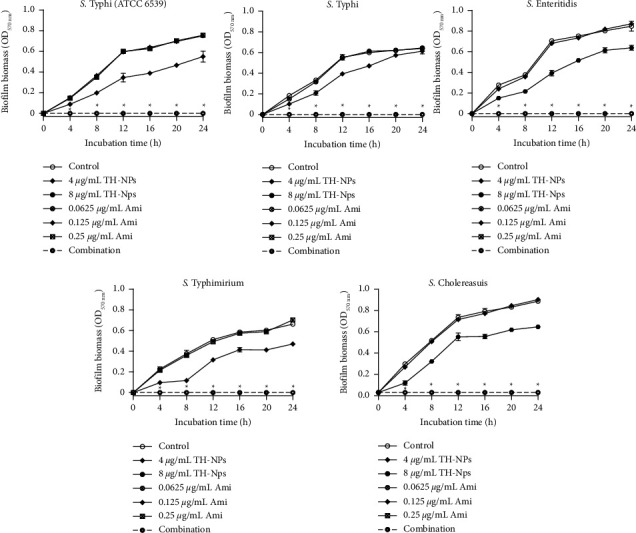
Kinetic study of TH-NPs and amikacin alone or in combination against biofilm biomass formation of *S. enterica* serovars. TH-NPs: thymol-loaded PLGA nanoparticles; Ami: amikacin. Data represent the average values and standard deviation of three independent experiments. ^*∗*^Significant difference between the combination and TH-NPs alone (*p* < 0.05).

The effects of TH-NPs and amikacin alone or in combination against the prevention of biofilm biomass formation of *S. enterica* serovars are shown in Figure 2. All curves have an upward trend. On the one hand, it appears that TH-NPs are more effective than antibiotics, and on the other hand, the combination of TH-NPs with amikacin completely inhibits biofilm formation in all strains.

**Figure 3:**
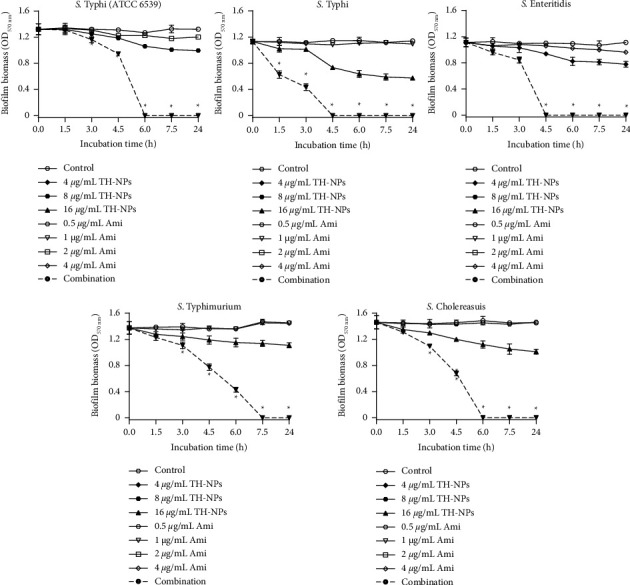
Kinetic study of TH-NPs alone or in combination with amikacin against disruption of preformed biofilm of *S. enterica* serovars. TH-NPs: thymol-loaded PLGA nanoparticles, Ami: amikacin. Data represent the average values and standard deviation of three independent experiments. ^*∗*^Significant difference between the combination and TH-NPs alone (*p* < 0.05).

Figure 3 shows the kinetic results of the biofilm biomass destruction of *Salmonella enterica* serovars treated with TH-NPs and amikacin alone or in combination. Compared to inhibition, where curves were ascending, here, curves are descending due to preformed biofilm. It should be noted that the combination effectively destroys the biofilm biomass of isolates at point times (4.5, 6, and 7.5 h). The biofilm disruption of *S*. Typhi was the most marked at 1.5 h with an optical density of 0.65.

### 3.6. Time-Kill Kinetic Assays

**Figure 4:**
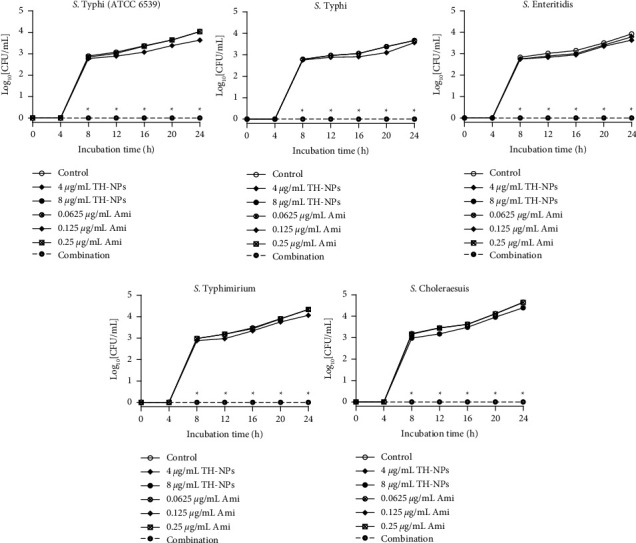
Time-kill kinetic assay of TH-NPs and amikacin alone or in combination against biofilm biomass formation of S. *enterica* serovars. TH-NPs: thymol-loaded PLGA nanoparticles; Ami: amikacin. Data represent the average values and standard deviation of three independent experiments. ^*∗*^Significant difference between the combination and TH-NPs alone (*p* < *0.05*).

The time-kill kinetic curves of TH-NPs and amikacin alone or in combination against biofilm biomass formation are shown in Figure 4. It was observed that, between 0 and 4 h, there was no growth. From 4 h, all the curves were ascending with log_10_ CFU/mL, ranging from 0 to 4.84. Taken individually, TH-NPs or amikacin did not significantly reduce the number of cells, while their combination induced significant (*p* ˂ 0.05) cell death.

**Figure 5:**
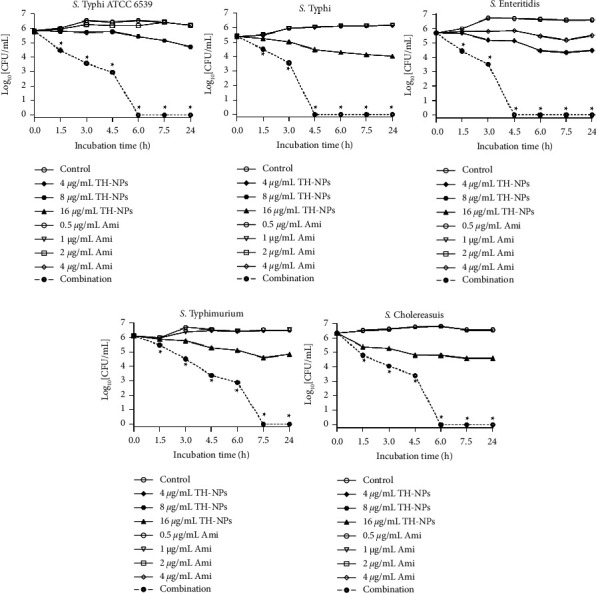
Time-kill kinetic assay of TH-NPs and amikacin alone or in combination against disruption of preformed biofilm of *S.enterica* serovars. TH-NPs: thymol-loaded PLGA nanoparticles; Ami: amikacin. Data represent the average values and standard deviation of three independent experiments. ^*∗*^Significant difference between the combination and TH-NPs alone (*p* < 0.05).

The results of the time-kill kinetic assay of TH-NPs and amikacin alone or in combination against disruption of preformed biofilm are plotted in Figure 5.

A perusal of Figure 5 reveals that the combination showed better activity than TH-NPs and amikacin only taken. At 3.0 h, we obtained a drop of at least 2 log_10_ CFU/mL compared to controls and compounds taken alone. Note that similar results were obtained during the kinetic study against biofilm biomass production. Here, at 6.0 h, cell death was ˃3 log_10_ CFU/mL reduction. Based on statistical analysis, (*p* ˂ 0.05), the combination compared to TH-NPs showed significant cell destruction.

## 4. Discussion

Antibiotic resistance is a serious challenge to the healthcare community both in developed and developing countries. The emergence and spread of multidrug-resistant pathogens have substantially threatened the current antibacterial therapy efficacy [30]. Indeed, the causes of antibiotic resistance are numerous, and several mechanisms have been proposed to explain the phenomenon of resistance within biofilms [31]. Recent studies have shown the ability of bacterial biofilms to survive high concentrations of antibiotics [32]. In addition, it is estimated that about 65% of all bacterial infections are associated with biofilms, and these include both device and nondevice-associated infections [32]. This necessitates a search for a new therapeutic agent with antibiofilm properties. In the last two decades, new approaches in preventing and eradicating biofilm have been widely developed and reported, including the potentiation of antibiotic activity by combination with bioactive natural products from plants [33].

Thymol (2-isopropyl-5-methylphenol) is a versatile molecule with a wide variety of practical applications such as medical, dentistry, veterinary, food, and agrochemicals. Its pharmacological applications have been most investigated and reported, focusing on its prominent antimicrobial, antioxidant, anti-inflammatory, and cicatrizing activities [34]. About antibacterial activities with MIC ranging from 64–128 *μ*g/mL and 4–8 *μ*g/mL, respectively, for thymol unloaded and TH-NPs on all four planktonic *Salmonella enterica* serovars and *S*. Typhi ATCC 6539, it is clear that TH-NPs are more efficient than free thymol. Indeed, the hydrophobic nature of thymol presents a challenge for microbial inhibition in aqueous media. However, PLGA produces acid by-products during the degradation process, which increases its hydrophobicity and enables it to partition into the lipids of the microbial cell membrane or to bind the hydrophobic regions of proteins [35]. Therefore, thymol could easily reach microorganisms after encapsulation in PLGA [36]. Moreover, recently, antibacterial activities of thymol-loaded PLGA microparticles have been tested against Gram-negative and Gram-positive bacteria via the colony-counting method, and it has been observed that antibacterial activity of thymol-loaded microparticles for both bacteria increased as the number of microparticles increased [17]. This can also be justified by the size (192.21 nm) and solubility of TH-NPs which can easily infiltrate the matrix, which acts as a barrier for many antibiotics [23], and pass through cell membranes [35]. These results are in agreement with those of Folle and collaborators, where the MICs of thymol nanoparticles were lower than those of nonencapsulated thymol on *Cutibacterium acnes* [37], and are also similar to those of Zhu et al., where TH-NPs had strong antibacterial activity against *Escherichia coli* and *Staphylococcus aureus* [17].

Concerning antibiofilm activity, with MBIC and MBEC ranging from 16 to 64 *μ*g/mL and 64 to 128 *μ*g/mL, respectively, for TH-NPs and unloaded thymol, they showed their ability to inhibit biofilm formation and eradicate *Salmonella* species mature biofilms.

Once again, the higher antibiofilm activity of TH-NPs may be due to their size and surface charge. TH-NPs' size substantially impacts their diffusion into the biofilm matrix, leading to more damage to biofilm cells. The nanoparticle's surface can be responsible for specific biofilm targeting by electrostatic interactions with the biofilm matrix, which favors the attachment of nanoparticles to the biofilm matrix and results in the release of the drug inside the biofilm [38]. In addition, the high surface area to volume ratio of nanoparticles allows for drug loading, which can result in strong antibiofilm efficacy [39]. The research found that thymol could inhibit biofilm formation and remove mature biofilms by inhibiting the production of polysaccharide intracellular adhesin (PIA) and the release of extracellular DNA (eDNA) [40]. In addition, previous studies have indicated that essential oils' components could inhibit the quorum-sensing system [41]. Yuan et al. reported antibiofilm activity of thymol toward methicillin-resistant*Staphylococcus aureus* [40]. Given the interesting activities of thymol, for clinical use, it would be important to pay attention to its toxicity. Note that, recently, a study has been performed to evaluate the acute and repeated 28-day oral dose toxicity of thyme essential oil in rats. Studies revealed moderate oral toxicity of this oil and suggested that the no-observed-adverse-effect level (NOAEL) is greater than 250 mg/kg/day [42]. This can sufficiently justify that thymol, a major component of thyme essential oil [43], is toxic at high concentrations. Nevertheless, natural compounds like thymol also present challenges for drug discovery, such as technical barriers to screening, isolation, solubility, stability, and volatility [44]. To overcome these problems, nanoparticles of natural products can be used. Nanoparticles of PLGA have been chosen in this study for their properties. PLGA is one of the most effective biodegradable polymeric nanoparticles. It has been approved by the United States Food and Drug Administration for use in drug delivery systems due to its controlled and sustained-release properties, low toxicity, biocompatibility with tissue [45], and biodegradability into lactic acid and glycolic acid, two monomers that are naturally produced under physiological conditions by several metabolic pathways [46]. In the previous study, based on the results obtained with aminoglycosides, amikacin was specially chosen for this study. Therefore, the synergistic effect of TH-NPs in combination with amikacin was investigated against biofilm formation by *S. enterica* serovars.

All combinations give synergetic effects on all strains. Some studies have explored the combination of synthetic drugs with essential oils, intending to evaluate and enhance antimicrobial efficacy [47]. For example, a synergistic effect was observed between norfloxacin and essential oil (and its main components) from *Pelargonium graveolens* [48], between gentamicin and the essential oil from *Croton zehntneri* [49], and between eugenol with antibiotics. To our knowledge, the antibiofilm capacity of TH-NPs and amikacin against microbial biofilms remains largely unknown. However, the antibacterial effect of thymol-loaded chitosan nanoparticles (TCNPs) has been reported against a broad spectrum of Gram-positive and Gram-negative pathogens. It is proposed that the charged groups in the polymer of nanoparticles and the moieties in thymol possibly interact with the negatively charged bacterial membrane, enhancing the killing efficiency of pathogens [45]. Note that the phenolic hydroxyl group of thymol increased its hydrophilic ability, which could help thymol dissolve in microbial membranes and damage them [17]. However, it is known that the ability of nanoparticles to attach and penetrate biofilm cells depends on their physicochemical properties such as size and morphology. TH-NPs would penetrate the biofilm and affect the quorum-sensing gene cascade, thereby hampering the cell-to-cell communication mechanism, which inhibits biofilm synthesis [44].

Moreover, it was shown that, at subinhibitory concentrations, thymol also reduces biofilm formation [40]. The results strongly suggest that a combination of TH-NPs with amikacin may be successfully used as an antibiofilm agent.

The synergistic antibiofilm activity and bactericidal action of TH-NPs in combination with amikacin were confirmed by the kinetic and time-kill assay of inhibition of biofilm biomass formation and eradication of preformed biofilm. Concerning the time-kill assay, a reduction greater than 3 log UFC/mL, compared to the control, which is equivalent to 99.9% killing of the inoculum bactericidal activity, was obtained [50].

This sufficiently shows the bactericidal activity of a combination of amikacin with TH-NPs. This study confirms that thymol is a potential effective source for medicinal chemists, which will take natural product-based antibiotic research to the next level [51].

## 5. Conclusion

This study showed that TH-NPs potentiate the antibiofilm activity of amikacin against the prevention and removal of *S*. *enterica* serovar biofilms. Synergistic and bactericidal effects obtained indicate that the combination of TH-NPs with amikacin is a promising approach for the development of novel antibacterial combination therapies against biofilm-associated infections. Further studies are under consideration to investigate the antibiofilm mechanism of action of TH-NPs and their combinations with antibiotics, especially the effect on quorum sensing. TH-NPs in combination with amikacin can be a promising alternative for the management of *Salmonella* biofilm.

## Figures and Tables

**Table 1:** Susceptibility of *S. enterica* planktonic cells against free thymol, thymol-loaded PLGA nanoparticles (TH-NPs), and the copolymer of PLGA.

Isolates	Susceptibility (*μ*g/mL)	Free thymol (*μ*g/mL)	TH-NPs (*μ*g/mL) (fold increase)	PLGA_c_
*S.* Typhi ATCC *6539*	MIC	64	4 [16]	>1024
	MBC	256	4 [64]	>1024

*S.* Enteritidis	MIC	64	8 [8]	>1024
	MBC	128	16 [8]	>1024

*S.* Typhi	MIC	64	8 [8]	>1024
	MBC	256	8 [32]	>1024

*S.* Typhimurium	MIC	128	8 [16]	>1024
	MBC	128	16 [8]	>1024

*S.* Choleraesuis	MIC	128	4 [32]	>1024
	MBC	256	8 [32]	>1024

MIC: minimum inhibitory concentration; MBC: minimum bacterial concentration; TH-NPs: thymol-loaded PLGA nanoparticles; [*x*]: a ratio between the concentration of free thymol and TH-NPs; PLGAc: unloaded nanoparticles.

**Table 2:** Minimum biofilm inhibitory concentration (MBIC) of amikacin and thymol-loaded PLGA and the fractional inhibitory concentration index (FICI) of combination against *S. enterica* serovars.

Isolates	MBEC (*μ*g/mL)				MBEC reduction fold		FICI (int.)
	Alone		Combined				
	Ami	TH-NPs	Ami	Thy	Ami	TH-NPs	
*S*. Typhi ATCC 6539	8	16	0.25	4	32	4	0.28 (S)
*S*. Typhi	8	32	0.0625	4	128	8	0.13 (S)
*S*. Enteritidis	4	64	0.125	8	32	8	0.28 (S)
*S*. Typhimurium	8	32	0.25	4	32	8	0.16 (S)
*S*. Choleraesuis	8	32	0.125	8	64	4	0.27 (S)

MBIC: minimum biofilm inhibitory concentration; Ami: amikacin; TH-NPs: thymol-loaded PLGA nanoparticles; FICI: fractional inhibitory index; INT: interpretation; S: synergy.

**Table 3:** Minimum biofilm eradication concentration (MBEC) of amikacin and thymol-loaded PLGA and the fractional inhibitory concentration index (FICI) of combination against *S*. *enterica* serovars.

Isolates	MBEC (*μ*g/mL)				MBEC reduction fold		FICI (int.)
	Alone		Combined				
	Ami	TH-NPs	Ami	Thy	Ami	TH-NPs	
*S*. Typhi ATCC 6539	128	64	2	8	64	8	0.14 (S)
*S*. Typhi	128	64	1	16	128	4	0.26 (S)
*S*. Enteritidis	128	128	4	4	32	32	0.06 (S)
*S*. Typhimurium	64	64	1	16	64	4	0.27 (S)
*S*. Choleraesuis	64	128	0,5	16	128	8	0.13 (S)

MBEC: minimum biofilm eradication concentration; Ami: amikacin; TH-NPs: thymol-loaded PLGA nanoparticles; FICI: fractional inhibitory index; INT: interpretation; S: synergy.

## Data Availability

The data used to support the findings of this study are available upon reasonable request from the corresponding author.
